# How do AI markings on screening mammograms correspond to cancer location? An informed review of 270 breast cancer cases in BreastScreen Norway

**DOI:** 10.1007/s00330-024-10662-2

**Published:** 2024-02-23

**Authors:** Henrik Wethe Koch, Marthe Larsen, Hauke Bartsch, Marit Almenning Martiniussen, Bodil Margrethe Styr, Siri Fagerheim, Ingfrid Helene Salvesen Haldorsen, Solveig Hofvind

**Affiliations:** 1 Department of Radiology, Stavanger University Hospital, Stavanger, Norway; 2 Faculty of Health Sciences, University of Stavanger, Stavanger, Norway; 3 Section for Breast Cancer Screening, Cancer Registry of Norway, Norwegian Institute of Public Health, P.O. Box 5313, 0304 Oslo, Norway; 4 Mohn Medical Imaging and Visualization Centre (MMIV), Department of Radiology, Haukeland University Hospital, Bergen, Norway; 5 Department of Radiology, Østfold Hospital Trust, Kalnes, Norway; 6 University of Oslo, Institute of Clinical Medicine, Oslo, Norway; 7 Section for Radiology, Department of Clinical Medicine, University of Bergen, Bergen, Norway; 8 Department of Health and Care Sciences, Faculty of Health Sciences, UiT The Arctic University of Norway, Tromsø, Norway

**Keywords:** Mammography, Breast neoplasm, Mammographic density, Artificial intelligence, Mass screening

## Abstract

**Objectives:**

To compare the location of AI markings on screening mammograms with cancer location on diagnostic mammograms, and to classify interval cancers with high AI score as false negative, minimal sign, or true negative.

**Methods:**

In a retrospective study from 2022, we compared the performance of an AI system with independent double reading according to cancer detection. We found 93% (880/949) of the screen-detected cancers, and 40% (122/305) of the interval cancers to have the highest AI risk score (AI score of 10). In this study, four breast radiologists reviewed mammograms from 126 randomly selected screen-detected cancers and all 120 interval cancers with an AI score of 10. The location of the AI marking was stated as correct/not correct in craniocaudal and mediolateral oblique view. Interval cancers with an AI score of 10 were classified as false negative, minimal sign significant/non-specific, or true negative.

**Results:**

All screen-detected cancers and 78% (93/120) of the interval cancers with an AI score of 10 were correctly located by the AI system. The AI markings matched in both views for 79% (100/126) of the screen-detected cancers and 22% (26/120) of the interval cancers. For interval cancers with an AI score of 10, 11% (13/120) were correctly located and classified as false negative, 10% (12/120) as minimal sign significant, 26% (31/120) as minimal sign non-specific, and 31% (37/120) as true negative.

**Conclusion:**

AI markings corresponded to cancer location for all screen-detected cancers and 78% of the interval cancers with high AI score, indicating a potential for reducing the number of interval cancers. However, it is uncertain whether interval cancers with subtle findings in only one view are actionable for recall in a true screening setting.

**Clinical relevance statement:**

In this study, AI markings corresponded to the location of the cancer in a high percentage of cases, indicating that the AI system accurately identifies the cancer location in mammograms with a high AI score.

**Key Points:**

*• All screen-detected and 78% of the interval cancers with high AI risk score (AI score of 10) had AI markings in one or two views corresponding to the location of the cancer on diagnostic images.*

*• Among all 120 interval cancers with an AI score of 10, 21% (25/120) were classified as a false negative or minimal sign significant and had AI markings matching the cancer location, suggesting they may be visible on prior screening.*

*• Most of the correctly located interval cancers matched only in one view, and the majority were classified as either true negative or minimal sign non-specific, indicating low potential for being detected earlier in a real screening setting.*

## Introduction

Breast cancer is the most common cancer type and cause of cancer death among women worldwide [1]. Early detection through mammographic screening is shown to significantly reduce mortality from the disease [2, 3] and is supported by international health authorities [4, 5]. However, the accuracy of mammographic interpretation varies, and 20–30% of the breast cancers are reported to be false negative in retrospective informed review studies [6–8]. Some of these cancers present as symptomatic interval cancers within the next screening round. Interval cancers tend to be less prognostically favorable compared to screen-detected cancers [9, 10], and the rate should be kept as low as possible.

Artificial intelligence (AI), including image-based deep learning, has emerged as a promising tool for improving the accuracy and efficiency of mammographic screening [11–16]. Several studies have shown that AI systems based on convolutional neural networks yield diagnostic performance at the near-expert radiologist level [12, 15–19].

In 2022, the research group conducted a retrospective study comparing the performance of a commercially available AI system to the outcome of independent double reading by radiologists according to cancer detection [20]. The AI system scored the examinations from 1 to 10, where 10 indicated a high risk of breast cancer. We found that 93% of 949 screen-detected cancers and 40% of 305 interval cancers were given a score of 10 by the AI system. However, we do not know if the AI markings on the screening mammograms correspond to the location of the tumor on the diagnostic mammograms. In general, there is limited knowledge of whether the AI markings are consistent with the location of the cancerous tumors, and whether the interval cancers identified by AI are actionable for recall or not. In a retrospective consensus review study from Sweden, 19% of all interval cancers with an AI score of 10 were correctly located by the AI system and classified as false negative or minimal signs [8]. Further studies are needed to ensure the validity of the AI markings, and to our knowledge, no studies have reviewed the location of the tumor for screen-detected cancers with different AI risk scores.

With the aim of contributing to filling the knowledge gaps related to this issue, we performed a retrospective informed consensus review of screening mammograms with AI markings and diagnostic mammograms of screen-detected and interval cancers. The main aim of the study was to assess whether markings given by the AI system on the screening mammograms corresponded to the location of the tumor on diagnostic mammograms for examinations with high AI risk scores (AI score of 10). Furthermore, we classified the interval cancers as false negative, minimal sign, or true negative based on mammographic findings on the screening mammograms. Lastly, we investigated screen-detected cancers with low AI risk scores (AI score of 1–7) to explore potential reasons for wrongly classifying these cases with low scores.

## Material and methods

The study was approved by the Regional Committee for Medical and Health Research Ethics (#13294). The data was disclosed with legal basis in the Cancer Registry Regulations of 21 December 2001 No. 47, Sect. 3–1 and the Personal Health Data Filing System Act Sect. 19 a to 19 h [21, 22].

A retrospective study of screening examinations performed in Rogaland, as a part of BreastScreen Norway 2010–2018 was the basis for this study [20]. BreastScreen Norway is a population-based screening program that started in 1996, administered by the Cancer Registry of Norway, and invites about 580,000 women aged 50–69 to two-view mammographic screening biennially [23]. All examinations are independently interpreted by two breast radiologists and each breast is given a score from 1 to 5 where 1 indicates normal findings, 2 probably benign, 3 intermediate suspicion, 4 probably malignant, and 5 high suspicion for malignancy. All examinations with a score of 2 or higher by either or both radiologists are discussed at a consensus meeting where a decision of recall is made. The attendance rate during 1996–2016 was 76%, recall 3.2%, screen-detected cancer 0.56%, and interval cancer 0.18% [24].

### Study sample

In the retrospective study, 13,896 screening examinations, including 949 screen-detected and 305 interval cancers performed in Rogaland County, were analyzed with a commercially available AI system (Transpara version 1.7.0, ScreenPoint Medical, Nijmegen). The system is Conformité Européenne (CE) marked and cleared by the U.S. Food and Drug Administration (FDA). The AI system scored all examinations from 1 to 10, where 1–7 indicated a low risk of breast cancer, 8–9 medium risk, and 10 high risk of breast cancer. For low-risk examinations, AI score of 1–7, AI markings were not available due to the specific set-up of the AI system in this project.

**Fig. 1:**
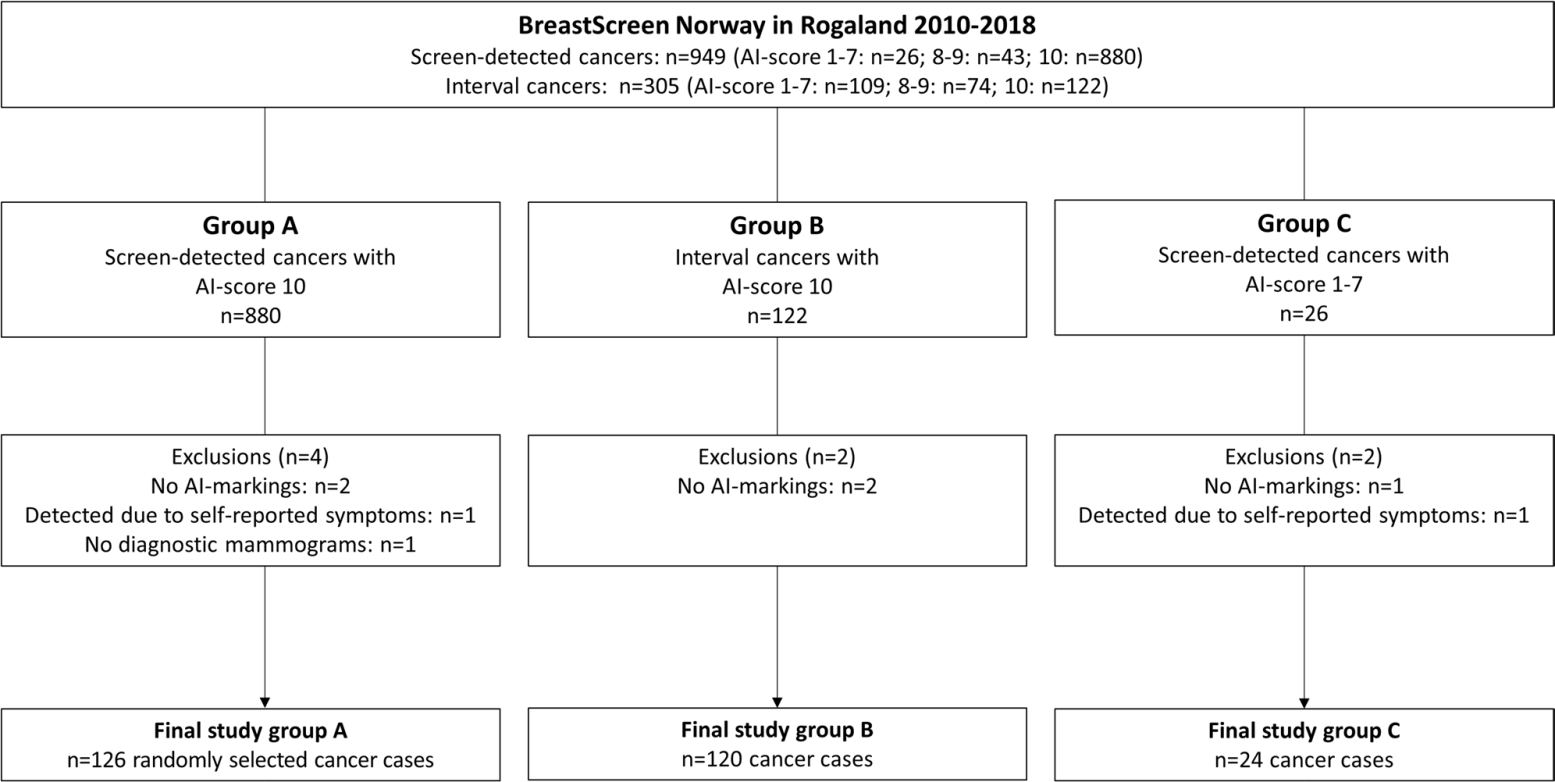
Flowchart of the study sample, including artificial intelligence score (AI score). An AI score of 10 indicates a high risk of breast cancer, 8–9 medium risk and 1–7 low risk of breast cancer

Among the 880 screen-detected cases with an AI score of 10, we randomly selected 130 for review (group A). Furthermore, all 122 interval cancers with an AI score of 10 (group B) and all 26 screen-detected cancers with an AI score of 1–7 (group C) were selected for review (Fig. 1). After excluding one cancer case without diagnostic mammograms available, two with no AI score available on the left breast, and one detected due to self-reported symptoms, the final study population in group A comprised 126 screen-detected cancers. Group B included 120 interval cancers after excluding two cases due to lack of AI markings on the mammograms, and group C included 24 screen-detected cancers after excluding one case detected due to self-reported symptoms and one case without AI score on the left breast where the cancer was located. For bilateral cancers in groups A and B, we only included the breast that was given an AI score of 10.

Screen-detected cancer was defined as breast cancer diagnosed after a recall for further assessment due to mammographic findings. Interval cancer was defined as breast cancer diagnosed after a negative screening result or more than 6 months after a false-positive screening result and within 24 months after screening. Breast cancer included ductal carcinoma in situ (DCIS) and invasive breast cancer.

### Informed mammographic review

A group of four breast radiologists (M.A.M., external, 7 years’ experience as breast radiologist; H.W.K., internal, 4 years’ experience; S.F., internal, 10 years’ experience; and B.S., internal, 30 years’ experience) and two secretaries performed the review. The informed review was consensus-based with screening and diagnostic mammograms available, in addition to markings and scores from the AI system on the screening mammograms. AI markings were available in the picture archiving and communication system (PACS) for cancer cases with an AI score of 10, but not for screen-detected cancers with an AI score of 1–7 as these were considered low-risk examinations.

For group A, the consensus recorded mammographic density (Breast Imaging Reporting and Data System [BI-RADS] a–d) and match/no match between location and views (craniocaudal [CC] and mediolateral oblique [MLO]) of the AI markings on screening mammograms and the tumor on diagnostic mammograms. Match/no match between the AI marking on the screening mammograms and cancer location on diagnostic mammograms was classified based on visual inspection by the four radiologists.

For group B, the consensus recorded mammographic density (BI-RADS a–d), match/no match between location and views of AI markings on screening mammograms and the tumor on diagnostic images, mammographic feature at screening and diagnostic mammograms, overall interpretation score (1–5) by the radiologists and classified the cases according to the EU guidelines from 2005; false negative, minimal signs (significant or non-specific findings), and true negative [5]. We recorded the review outcome into the following four categories: false negative (obvious visible abnormal findings on priors), minimal sign significant (subtle findings at the cancer site that would not necessarily be regarded as warranting a recall), minimal sign non-specific (non-specific findings at the cancer site with a recall not considered probable), and true negative (no visible abnormal findings on the screening mammograms).

Mammographic features were also recorded and classified according to a modified BI-RADS system [25], as mass, spiculated mass, calcifications, asymmetry [26], architectural distortion [27], and density with calcifications.

For group C, the consensus recorded mammographic density (BI-RADS a–d), overall score by the radiologists (1–5), and mammographic features at screening. Mammographic features of the cancers were compared with mammographic features at prior examinations, to decide whether the finding was a new or a developing lesion.

### Statistical analysis

Analyses were descriptive and presented as frequencies and percentages. Results were stratified by view and classification (false negative, minimal sign (total and significant/non-specific), and true negative). Results concerning mammographic density and features were presented in the figures. All analyses were performed with Stata (StataCorp. 2021. Stata Statistical Software: Release 17. StataCorp LLC).

## Results

### Group A—screen-detected cancers with an AI score of 10

All 126 screen-detected cancers with an AI score of 10 were correctly located by the AI system in either CC, MLO, or both views (Table 1). We found 79% (100/126) of the cases to match in both CC and MLO view, while 10% (12/126) matched solely in CC and 11% (14/126) only in MLO. In total, 96% (121/126) of the screen-detected cancers were classified as BI-RADS mammographic density a or b, and 67% (84/126) as density b.

**Table 1:** Match between artificial intelligence (AI) markings on screening mammograms and cancer location on diagnostic mammograms by BI-RADS mammographic density (a–d) and mammography view. Percentages are calculated based on the total number in each density group or total

	Correct location for AI markings on the screening mammograms and cancer on diagnostic mammograms			
	CC and MLO *n* (%)	CC only *n* (%)	MLO only *n* (%)	All combinations *n* (%)
Group A – screen-detected cancers (*n* = 126)				
Density a (*n* = 37)	28 (76%)	4 (11%)	5 (14%)	37 (100%)
Density b (*n* = 84)	68 (81%)	8 (10%)	8 (10%)	84 (100%)
Density c (*n* = 5)	4 (80%)	-	1 (20%)	5 (100%)
Density d (*n* = 0)	-	-	-	-
Total (*n* = 126)	100 (79%)	12 (10%)	14 (11%)	126 (100%)
Group B – Interval cancers (*n* = 120)				
Density a (*n* = 2)	-	-	1 (50%)	1 (50%)
Density b (*n* = 47)	11 (23%)	15 (32%)	10 (21%)	36 (77%)
Density c (*n* = 66)	14 (21%)	19 (29%)	14 (21%)	54 (82%)
Density d (*n* = 5)	1 (20%)	-	1 (20%)	2 (40%)
Total (*n* = 120)	26 (22%)	36 (30%)	31 (26%)	93 (78%)

*CC*, craniocaudal view; *MLO*, mediolateral oblique view

### Group B—interval cancers with an AI score of 10

A total of 78% (93/120) of interval cancers with an AI score of 10 were correctly located by the AI system in either CC, MLO, or both views (Table 1). We found 22% (26/120) to match in both views, 30% (36/120) in CC, and 26% (31/120) in MLO. A vast majority of the interval cancers, 94% (113/120), were classified as density b or c (55% (66/120) as density c).

**Fig. 2:**
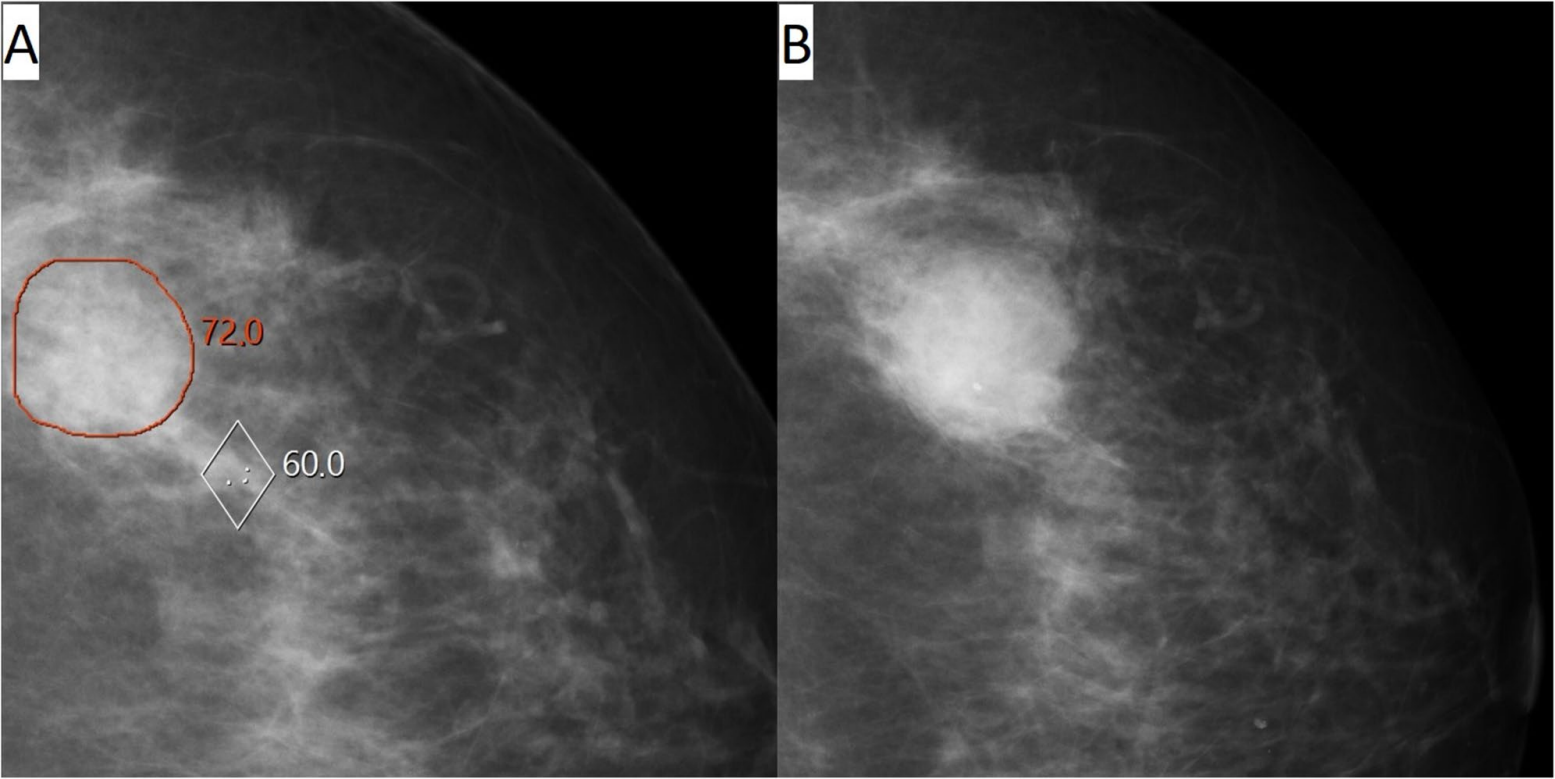
Woman, 64 years old with interval cancer diagnosed 560 days after screening. An artificial intelligence (AI) score of 10 and AI markings on the screening mammogram (**A**) matching the location of the tumor on diagnostic images (**B**). Classified as “false negative” in an informed review by four breast radiologists with AI score and diagnostic images available

**Fig. 3:**
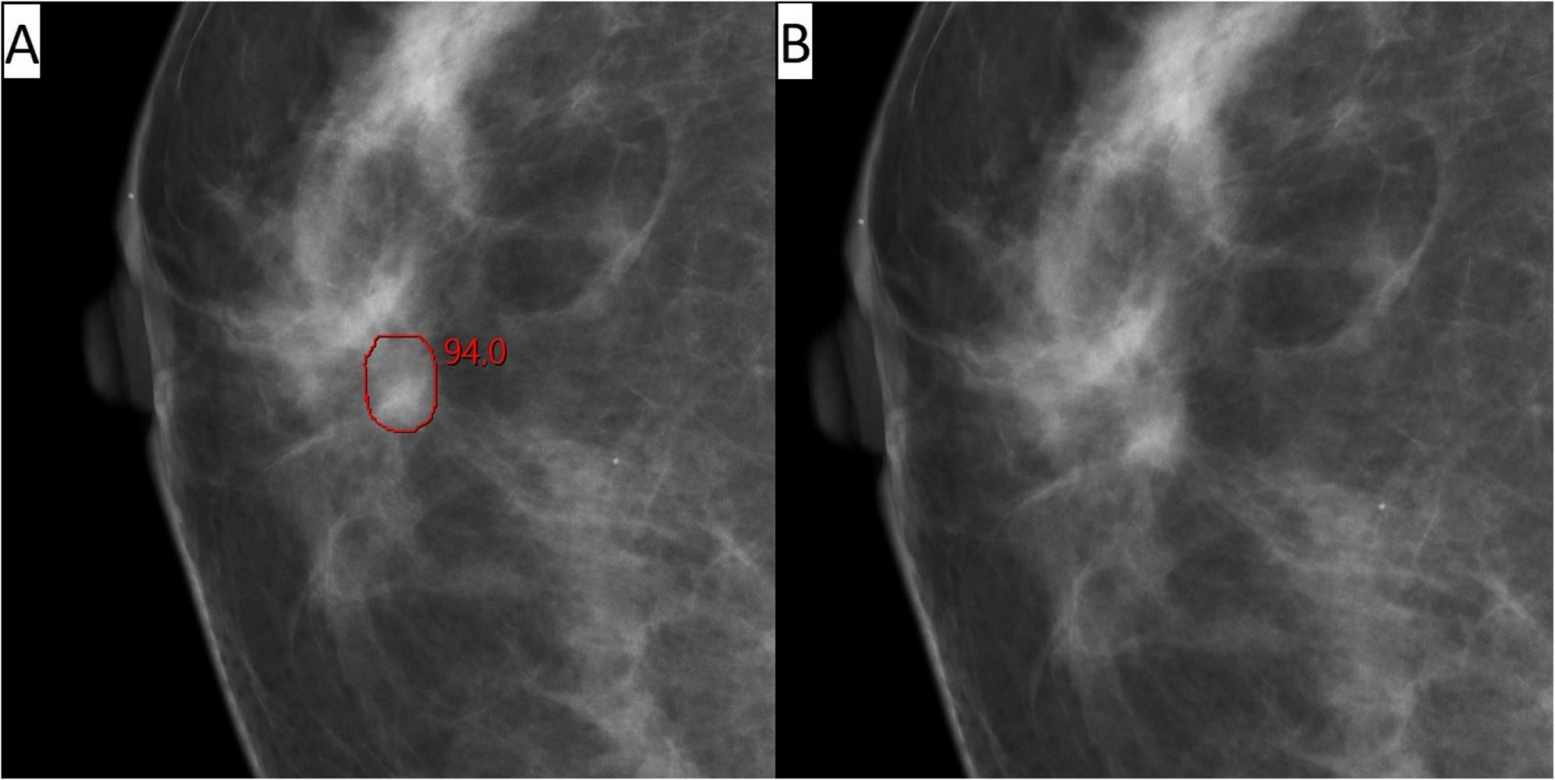
Woman, 59 years old with interval cancer diagnosed 219 days after screening. An artificial intelligence (AI) score of 10 and AI markings on the screening mammogram (**A**) matching the location of the tumor on diagnostic images (**B**). Classified as “minimal sign significant” in an informed review by four breast radiologists with AI score and diagnostic images available

**Fig. 4:**
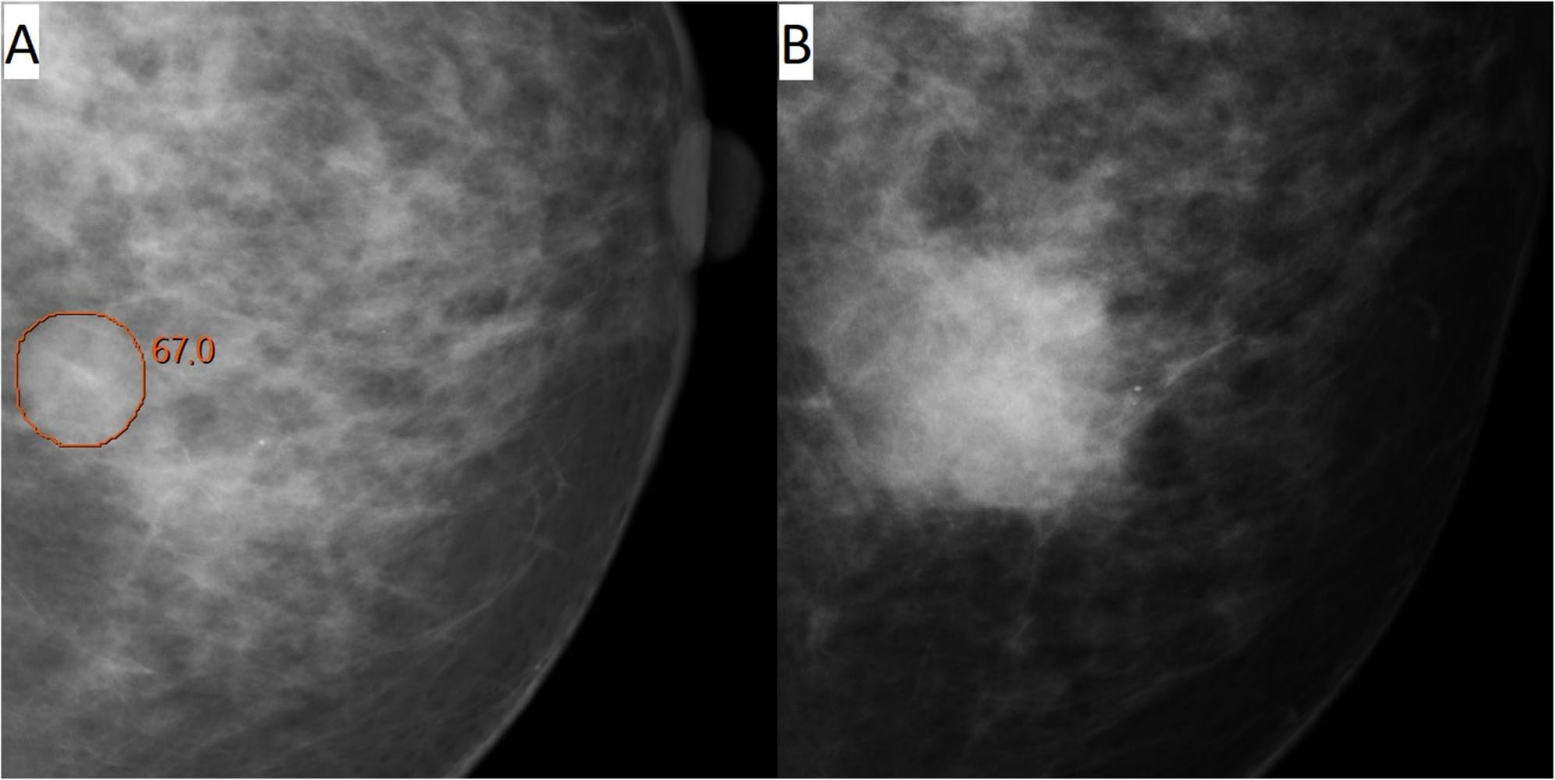
Woman, 55 years old with interval cancer diagnosed 200 days after screening. An artificial intelligence (AI) score of 10 and AI markings on the screening mammogram (**A**) matching the location of the tumor on diagnostic images (**B**). Classified as “minimal sign non-specific” in an informed review by four breast radiologists with AI score and diagnostic images available

**Fig. 5:**
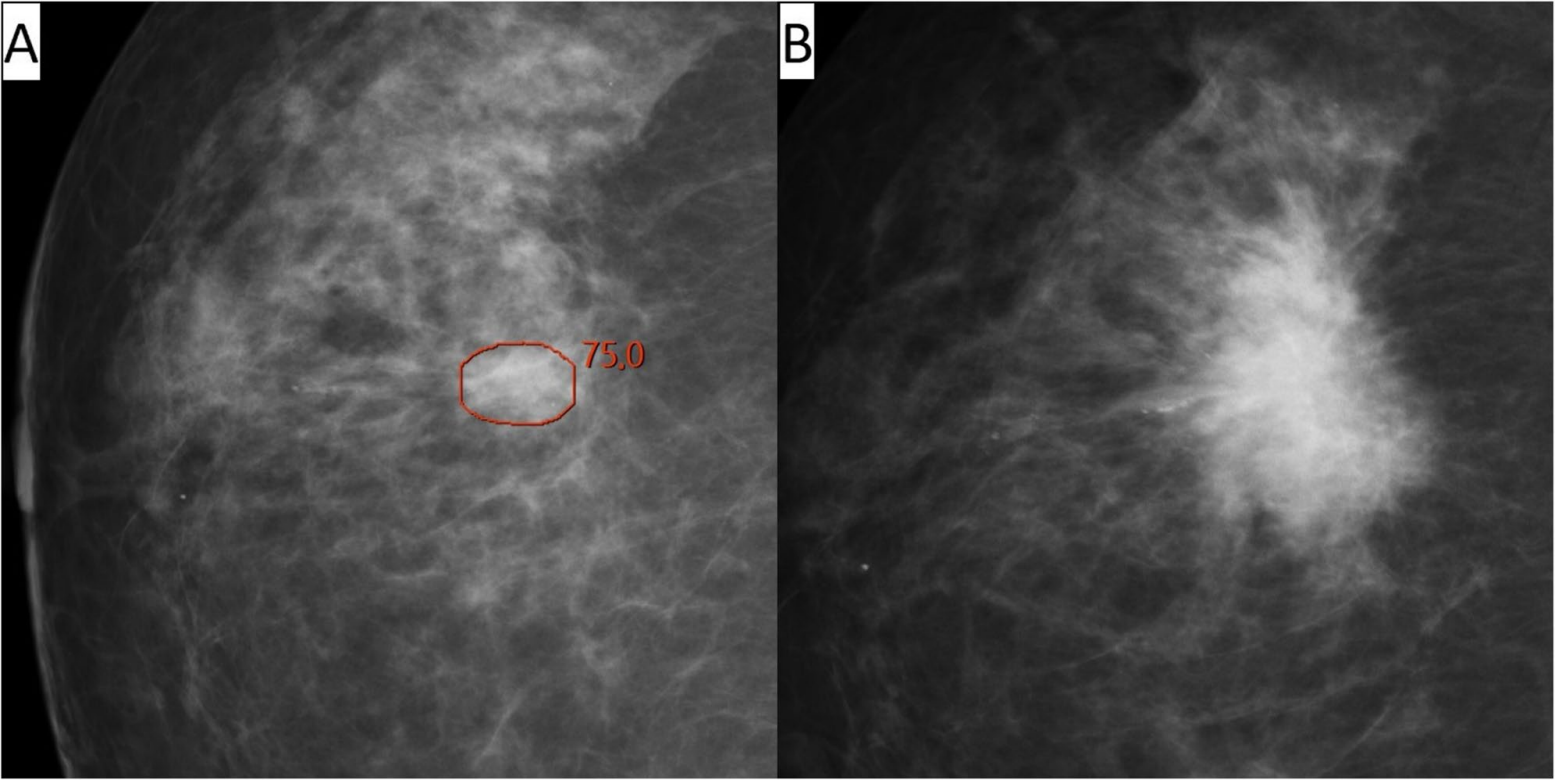
Woman, 69 years old with interval cancer diagnosed 623 days after screening. An artificial intelligence (AI) score of 10 and AI markings on the screening mammogram (**A**) matching the location of the tumor on diagnostic images (**B**). Classified as “true negative” in an informed review by four breast radiologists with AI score and diagnostic images available

Among all 120 interval cancers with an AI score of 10, 11% (13/120) were correctly located and classified as false negative, 36% (43/120) were correctly located and classified as minimal sign (10% (12/120) as minimal sign significant and 26% (31/120) as minimal sign non-specific), and 31% (37/120) were correctly located and classified as true negative (Table 2, Figs. 2, 3, 4, and 5). Among the 25 correctly located interval cancers classified as either false negative (*n* = 13) or minimal sign significant (*n* = 12), 48% (12/25) matched in both views. For the 68 correctly located interval cancers classified as minimal sign non-specific (*n* = 31) or true negative (*n* = 37), 21% (14/68) matched in both views.

**Table 2:** Results of review classifications of interval cancers with artificial intelligence (AI) score 10 (group B) and correct/not correct location of the AI markings, classified as false negative, minimal sign and true negative. Percentages are calculated with total number of interval cancers with an AI score of 10 (*n* = 120) as denominator

Classifications of interval cancers with an AI score of 10	Correct location of the AI marking	Not correct location of the AI marking	Total
False negative	13 (11%)	0 (-)	13 (11%)
Minimal sign significant	12 (10%)	1 (1%)	13 (11%)
Minimal sign non-specific	31 (26%)	7 (6%)	38 (32%)
True negative	37 (31%)	19 (16%)	56 (47%)
Total	93 (78%)	27 (23%)	120 (100%)

Among the 27 interval cancers where the AI markings did not match the location of the tumor, 70% (19/27) were classified as true negative (Table 2).

**Fig. 6:**
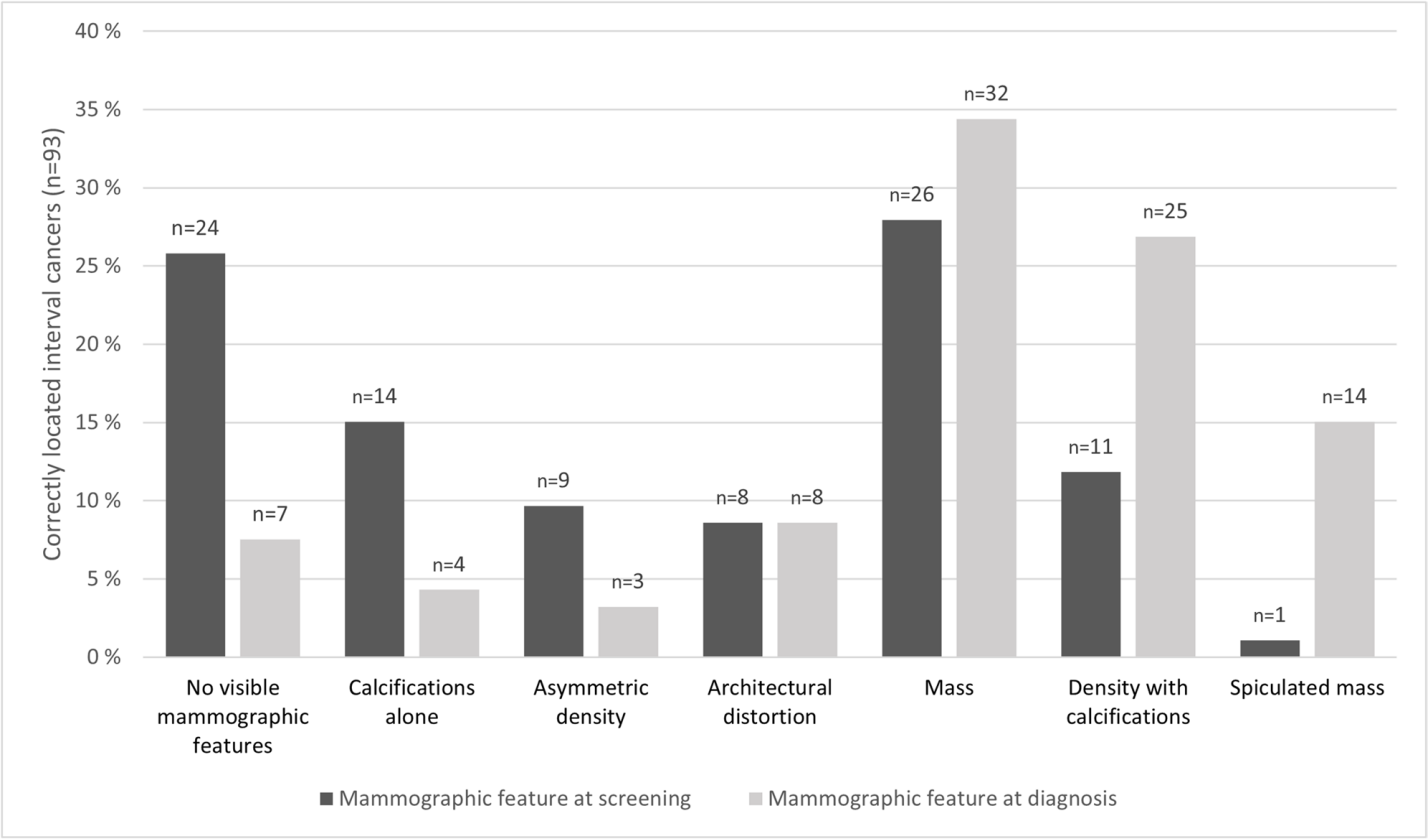
Mammographic features at screening and diagnosis for interval cancers with an artificial intelligence (AI) score of 10 and AI markings matching the location of the cancer (*n* = 93)

We did not find any visible features on the screening mammograms for 26% (24/93) of the interval cancer cases with a correct location of the AI marking, while 8% (7/93) were considered to be without any visible features on the diagnostic mammogram (Fig. 6). The number of cases with no visible mammographic features at the time of screening (*n* = 24) was lower than the number of cases classified as true negative (*n* = 37) as 13 cases with visible features were considered negative by the reviewers. At the time of screening, mass (28% [26/93]) and calcifications alone (15% [14/93]) were the most common mammographic features for these cases. At the time of diagnosis, mass and density with calcifications were the most frequent features, observed in 34% (32/93) and 27% (25/93), respectively. When comparing the distribution of mammographic features for interval cancers at the time of diagnosis versus the time of screening, the proportion with asymmetry and calcifications decreased the most, while the proportion with spiculated mass and density with calcifications increased the most. The median time from screening to diagnosis of interval cancer was 428 days (IQR: 250–586).

### Group C—screen-detected cancers with an AI score of 1–7

Group C included 24 screen-detected cancers with an AI score of 1–7, three with an AI score of 1, two with an AI score of 2, three with an AI score of 4, six with an AI score of 5, three with an AI score of 6, and seven with an AI score of 7. We found 25% (6/24) of the cases to be BI-RADS mammographic density a, 50% (12/24) as b, 25% (6/24) as c, and none in density group d.

Furthermore, 71% (17/24) of the cases were classified as “mass.” When compared to prior mammograms, 58% (14/24) were considered new lesions and 21% (5/24) as developing asymmetry. The reviewing radiologists’ interpretation score was 1 for 21% (5/24) of the cases, 2 for 58% (14/24), 3 for 17% (4/24), and 4 for 4% (1/24). None had an interpretation score of 5. The five cancer cases with an interpretation score of 3 or 4 were considered visible for the radiologists and thus false negatives by the AI system.

## Discussion

In this study, we found that all screen-detected cancers and 78% (93/120) of the interval cancers with an AI score of 10 had AI markings on the screening mammograms matching the location of the cancer on diagnostic images. Among all interval cancers with an AI score of 10, 21% (25/120) had AI markings on the correct location and were classified as either false negative or minimal sign significant in an informed consensus review by the four breast radiologists in this study, suggesting that they may be visible at prior screening.

Our results indicate that the AI system can be trusted when it comes to the correct marking of screen-detected tumors with high AI scores. For the screen-detected cases, the majority (79% [100/126]) of the tumors were marked correctly in both CC and MLO, as opposed to interval cancers, where only 22% (26/120) matched in both views. It is not expected to observe similarly accurate AI markings for interval cancers, as many of these cancers may be fast-growing and thus not present or visible on prior mammograms. Our findings indicate that AI may potentially contribute to reducing the interval cancer rate by increasing the detection at mammography screening, but also clearly demonstrate that interval cancer cases represent a challenge for AI as well as for radiologists.

In theory and only based on AI score 10 and correctly marked location on the screening mammograms, AI-based screening could yield about a 30% (40%*78%) reduction in the total number of interval cancers, given that all potential interval cancers with AI score 10 will be recalled and diagnosed as screen-detected instead of interval cancers. However, most of the correctly located interval cancers matched only in one view, and the majority were classified as either true negative or minimal sign non-specific, indicating low potential for being detected earlier in a screening setting using radiologists and AI support.

In our published paper from 2022, 40% (122/305) of the interval cancers had an AI score of 10 [20] which was higher than 33% (143/429) reported in a Swedish review study using an older version of the same AI system [8]. Despite reporting a higher proportion of cases with an AI score of 10, we found a slightly lower proportion of interval cancer cases with the correct location and at least minimal signs of malignancy (i.e., not classified as true negative). The Swedish study reported that 19% (83/429) of all the interval cancers were correctly located and had at least minimal signs of malignancy. The corresponding number in our study was 18% (56/305), showing that the two studies are comparable with regards to the overall potential of AI to detect interval cancers with visible findings.

Although it is remarkable that the AI system was able to identify cancers that were occult even to the trained human eye, it is unlikely that these cases would be actionable for recall in a real screening setting, especially when considering the expected high number of false positive AI markings. This raises important questions regarding the practical usefulness of AI in screening. How are radiologists supposed to apprehend AI findings that are occult or considered negative or benign? Selecting all women with an AI score of 10 for recall regardless of human perception and interpretation of visual findings would yield an unacceptably high recall rate, roughly 2–4 times higher than the current rate in BreastScreen Norway [23, 24]. On the other hand, deselecting women with high AI scores due to inconsistency between the AI system and the radiologist is likely to result in false negative cancers, which may also have legal implications. Several strategies can possibly deal with this problem, for example, shorter screening intervals and/or other screening techniques for women with a high AI score. However, such strategies may involve increased costs due to increased workload for radiologists and frequent screening schemes. Robust and large prospective studies are clearly needed in order to define a cost-effective optimal setup for handling and follow-up of patients based on their AI scores.

When comparing the distribution of mammographic features for interval cancers at the time of diagnosis versus the time of screening, asymmetry, and calcifications decreased the most, while spiculated mass and density with calcifications increased the most. These observations suggest that AI-based screening could potentially aid in detecting small cancers with asymmetries and calcifications before they develop into larger masses. However, it is uncertain whether these cases with subtle findings are actionable for recall in a true screening setting.

Nineteen of the 24 screen-detected cancers with low AI risk score (Group C) had a low interpretation score, 1 or 2, by the radiologists, indicating that the lesions were not suspicious of malignancy. The cases were likely selected for further assessment due to a newcoming feature observed when compared with prior screening mammograms. Worth noting is that many of these cases represented new lesions or developing asymmetries in fatty breasts, where the sensitivity of the radiologists is known to be high [28–30]. Our observations highlight the importance of developing AI algorithms capable of using prior screening mammograms for comparison. Before such systems are available, AI systems used in a stand-alone fashion without a human reader run the risk of missing cancers that would otherwise be unacceptable for radiologists to miss. On the other hand, using AI as a stand-alone second reader or as decision support seems viable, and is supported by our findings that most screen-detected cancers, and a substantial number of interval cancers, were identified by the AI system.

The strengths of our study are the large number of cancer cases in the initial study, and that image data was merged with screening data from the Cancer Registry of Norway, which is close to 100% complete for breast cancer [31]. The generalizability of our findings is, however, subject to certain limitations—mostly related to the retrospective nature of this study. Reviewers had access to diagnostic images and AI scores during the review, and there are inherent limitations of an informed consensus review approach. Furthermore, since we selected a random subsample of screen-detected cancers with an AI score of 10 from all 880 such cases, we expected the subsample to be representative of the entire sample, and no difference in the results if the same radiologists had reviewed all 880 cases. However, other studies using other screening populations and radiologists might have different results.

In conclusion, AI markings corresponded to the location of the cancers in a high percentage of cases with an AI score of 10. However, the true potential for earlier detection of interval cancers may be somewhat reduced in a real screening setting, as most interval cancers had subtle or no visible findings and would likely not be recalled by human readers, due to the risk of increasing the rate of false positive screening results.

## Abbreviations

AI: Artificial intelligence
BI-RADS: Breast Imaging Reporting and Data System
CC: Craniocaudal view
DCIS: Ductal carcinoma in situ
MLO: Mediolateral oblique view
PACS: Picture Archiving and Communication System

## Declarations

### Guarantor

The scientific guarantor of this publication is Solveig Hofvind.

### Statistics and biometry

One of the authors has significant statistical expertise, but no complex statistical methods were necessary for this paper.

### Study subjects or cohorts overlap

A retrospective study of screening examinations performed in Rogaland, as a part of BreastScreen Norway 2010–2018 was the basis for this study: Koch HW, Larsen M, Bartsch H, Kurz KD, Hofvind S (2023) Artificial intelligence in BreastScreen Norway: a retrospective analysis of a cancer-enriched sample including 1254 breast cancer cases. Eur Radiol 33:3735–3743. The submitted study is a follow-up study of the paper in Eur Radiol, aimed at providing a deeper understanding of the validity of the AI markings on screening mammograms.

### Methodology

• an informed review of screening and diagnostic mammograms

• retrospective

• registry study / observational study

• performed at one institution

## References

[1] Sung H, Ferlay J, Siegel RL et al (2021) Global Cancer Statistics 2020: GLOBOCAN estimates of incidence and mortality worldwide for 36 cancers in 185 countries. CA Cancer J Clin 71:209–249 33538338 10.3322/caac.21660

[2] Marmot MG, Altman DG, Cameron DA, Dewar JA, Thompson SG, Wilcox M (2013) The benefits and harms of breast cancer screening: an independent review. Br J Cancer 108:2205–2240 23744281 10.1038/bjc.2013.177 PMC3693450

[3] Lauby-Secretan B, Scoccianti C, Loomis D et al (2015) Breast-cancer screening–viewpoint of the IARC Working Group. N Engl J Med 372:2353–2358 26039523 10.1056/NEJMsr1504363

[4] International Agency for Research on Cancer (2016) IARC Handbooks of Cancer Prevention, vol 15. Breast Cancer Screening, 2nd edn. IARC Press, Lyon

[5] European Commission Initiative on Breast Cancer (2024) European guidelines on breast cancer screening and diagnosis. Available via https://healthcare-quality.jrc.ec.europa.eu/en/ecibc/european-breast-cancer-guidelines. Accessed 12 Feb 2024

[6] Hovda T, Hoff SR, Larsen M, Romundstad L, Sahlberg KK, Hofvind S (2021) True and missed interval cancer in organized mammographic screening: a retrospective review study of diagnostic and prior screening mammograms. Acad Radiol. 10.1016/j.acra.2021.03.022 33926794 10.1016/j.acra.2021.03.022

[7] Hoff SR, Samset JH, Abrahamsen AL, Vigeland E, Klepp O, Hofvind S (2011) Missed and true interval and screen-detected breast cancers in a population based screening program. Acad Radiol 18:454–460 21216632 10.1016/j.acra.2010.11.014

[8] Lang K, Hofvind S, Rodriguez-Ruiz A, Andersson I (2021) Can artificial intelligence reduce the interval cancer rate in mammography screening? Eur Radiol. 10.1007/s00330-021-07686-3 33486604 10.1007/s00330-021-07686-3 PMC8270858

[9] Meshkat B, Prichard RS, Al-Hilli Z et al (2015) A comparison of clinical-pathological characteristics between symptomatic and interval breast cancer. Breast 24:278–282 25771080 10.1016/j.breast.2015.02.032

[10] Houssami N, Hunter K (2017) The epidemiology, radiology and biological characteristics of interval breast cancers in population mammography screening. NPJ Breast Cancer 3:12 28649652 10.1038/s41523-017-0014-x PMC5460204

[11] Raya-Povedano JL, Romero-Martin S, Elias-Cabot E, Gubern-Merida A, Rodriguez-Ruiz A, Alvarez-Benito M (2021) AI-based strategies to reduce workload in breast cancer screening with mammography and tomosynthesis: a retrospective evaluation. Radiology 300:57–65 33944627 10.1148/radiol.2021203555

[12] Lång K, Dustler M, Dahlblom V, Åkesson A, Andersson I, Zackrisson S (2021) Identifying normal mammograms in a large screening population using artificial intelligence. Eur Radiol 31:1687–1692 32876835 10.1007/s00330-020-07165-1 PMC7880910

[13] Rodriguez-Ruiz A, Lang K, Gubern-Merida A et al (2019) Can we reduce the workload of mammographic screening by automatic identification of normal exams with artificial intelligence? A feasibility study. Eur Radiol 29:4825–4832 30993432 10.1007/s00330-019-06186-9 PMC6682851

[14] Dembrower K, Wahlin E, Liu Y et al (2020) Effect of artificial intelligence-based triaging of breast cancer screening mammograms on cancer detection and radiologist workload: a retrospective simulation study. Lancet Digit Health 2:e468–e474 33328114 10.1016/S2589-7500(20)30185-0

[15] Lang K, Josefsson V, Larsson AM et al (2023) Artificial intelligence-supported screen reading versus standard double reading in the Mammography Screening with Artificial Intelligence trial (MASAI): a clinical safety analysis of a randomised, controlled, non-inferiority, single-blinded, screening accuracy study. Lancet Oncol 24:936–944 37541274 10.1016/S1470-2045(23)00298-X

[16] Salim M, Dembrower K, Eklund M, Smith K, Strand F (2023) Differences and similarities in false interpretations by AI CAD and radiologists in screening mammography. Br J Radiol. 10.1259/bjr.20230210:20230210 37660400 10.1259/bjr.20230210:20230210 PMC10607417

[17] Larsen M, Aglen CF, Lee CI et al (2022) Artificial Intelligence Evaluation of 122 969 Mammography Examinations from a Population-based Screening Program. Radiology 303:502–511 35348377 10.1148/radiol.212381 PMC9131175

[18] Rodriguez-Ruiz A, Lang K, Gubern-Merida A et al (2019) Stand-alone artificial intelligence for breast cancer detection in mammography: comparison with 101 radiologists. J Natl Cancer Inst 111:916–922 30834436 10.1093/jnci/djy222 PMC6748773

[19] Freeman K, Geppert J, Stinton C et al (2021) Use of artificial intelligence for image analysis in breast cancer screening programmes: systematic review of test accuracy. BMJ 374:n1872 34470740 10.1136/bmj.n1872 PMC8409323

[20] Koch HW, Larsen M, Bartsch H, Kurz KD, Hofvind S (2023) Artificial intelligence in BreastScreen Norway: a retrospective analysis of a cancer-enriched sample including 1254 breast cancer cases. Eur Radiol 33:3735–3743 36917260 10.1007/s00330-023-09461-y PMC10121532

[21] Lovdata (2001) Forskrift om innsamling og behandling av helseopplysninger i Kreftregisteret (Kreftregisterforskriften). Available via https://lovdata.no/dokument/LTI/forskrift/2001-12-21-1477. Accessed 12 Feb 2024

[22] Lovdata (2014) Lov om helseregistre og behandling av helseopplysninger (Helseregisterloven). Available via https://lovdata.no/dokument/LTI/lov/2014-06-20-43. Accessed 12 Feb 2024

[23] Bjørnson EW, Holen ÅS, Sagstad S et al (2022) BreastScreen Norway: 25 years of organized screening. Oslo: Cancer Registry of Norway. Available via https://www.kreftregisteret.no/globalassets/mammografiprogrammet/rapporter-og-publikasjoner/2022-25-arsrapport_webversjon.pdf. Accessed 12 Feb 2024

[24] Hofvind S, Tsuruda K, Mangerud G, Ertzaas AK (2017) The Norwegian breast cancer screening program, 1996–2016: celebrating 20 years of organized mammographic screening. Oslo: Cancer Registry of Norway. Available via https://www.kreftregisteret.no/globalassets/mammografiprogrammet/rapporter-og-publikasjoner/2022-25-arsrapport_webversjon.pdf. Accessed 12 Feb 2024

[25] Sickles EA, D’Orsi CJ, Bassett LW, et al (2013) ACR BI-RADS® Mammography. In: ACR BI-RADS® Atlas, Breast Imaging Reporting and Data System. Reston, VA, American College of Radiology

[26] Barazi H, Gunduru M (2023) Mammography BI RADS grading. In: StatPearls [Internet], Treasure Island (FL), StatPearls Publishing, Available via https://www.ncbi.nlm.nih.gov/books/NBK539816/. Accessed 12 Feb 2024 30969638

[27] D’Orsi CJ, Newell MS (2007) BI-RADS decoded: detailed guidance on potentially confusing issues. Radiol Clin North Am 45(751–763):v 17888766 10.1016/j.rcl.2007.06.003

[28] Boyd NF, Guo H, Martin LJ et al (2007) Mammographic density and the risk and detection of breast cancer. N Engl J Med 356:227–236 17229950 10.1056/NEJMoa062790

[29] Lauritzen AD, Rodriguez-Ruiz A, von Euler-Chelpin MC et al (2022) An artificial intelligence-based mammography screening protocol for breast cancer: outcome and radiologist workload. Radiology. 10.1148/radiol.210948:210948 35438561 10.1148/radiol.210948:210948

[30] Posso M, Louro J, Sanchez M et al (2019) Mammographic breast density: How it affects performance indicators in screening programmes? Eur J Radiol 110:81–87 30599878 10.1016/j.ejrad.2018.11.012

[31] Larsen IK, Smastuen M, Johannesen TB et al (2009) Data quality at the Cancer Registry of Norway: an overview of comparability, completeness, validity and timeliness. Eur J Cancer 45:1218–1231 19091545 10.1016/j.ejca.2008.10.037

